# Geometry-enhanced graph neural networks accelerate circRNA therapeutic target discovery

**DOI:** 10.3389/fgene.2025.1633391

**Published:** 2025-07-07

**Authors:** Zhen Li, Mingming Qi, Juyuan Huang, Wei Zhang, Xu Tan, Yifan Chen

**Affiliations:** ^1^ School of Artificial Intelligence, Shenzhen Institute of Information Technology, Shenzhen, China; ^2^ School of Data Science and Artificial Intelligence, Wenzhou University of Technology, Wenzhou, China; ^3^ Department of Gynecology, Zhongnan Hospital of Wuhan University, Wuhan, China; ^4^ School of Information Engineering, Changsha Medical University, Changsha, China

**Keywords:** drug development, circRNA therapeutic targets, geometric graph representation, biomarker discovery, circRNA-drug network

## Abstract

Circular RNAs (circRNAs) play pivotal roles in various biological processes and disease progression, particularly in modulating drug responses and resistance mechanisms. Accurate prediction of circRNA-drug associations (CDAs) is essential for biomarker discovery and the advancement of therapeutic strategies. Although several computational approaches have been proposed for identifying novel circRNA therapeutic targets, their performance is often limited by inadequate modeling of higher-order geometric information within circRNA-drug interaction networks. To overcome these challenges, we propose G2CDA, a geometric graph representation learning framework specifically designed to enhance the identification of CDAs and facilitate therapeutic target discovery. G2CDA introduces torsion-based geometric encoding into the message propagation process of the circRNA-drug network. For each potential association, we construct local simplicial complexes, extract their geometric features, and integrate these features as adaptive weights during message propagation and aggregation. This design promotes a richer understanding of local topological structures, thereby improving the robustness and expressiveness of learned circRNA and drug representations. Extensive benchmark evaluations on public datasets demonstrate that G2CDA outperforms state-of-the-art CDA prediction models, particularly in identifying novel associations. Case studies further confirm its effectiveness by uncovering potential drug interactions with the ALDH3A2 and ANXA2 biomarkers. Collectively, G2CDA provides a robust and interpretable framework for accelerating circRNA-based therapeutic target discovery and streamlining drug development pipelines. Our code are archived in: https://github.com/lizhen5000/G2CDA.

## Introduction

Circular RNAs (circRNAs) are highly stable endogenous non-coding RNAs resistant to nuclease degradation due to their covalently closed loop structure (Geng et al., 2020). Numerous studies indicate that circRNAs play crucial roles in various biological processes, such as transcriptional regulation, miRNA sponge effects, and protein interaction modulation (Zang et al., 2020). In disease research, particularly in cancer, circRNA aberrant expression is closely linked to tumorigenesis, progression, and drug resistance (Xu et al., 2020). Mounting evidence indicates that circRNAs play a crucial role in drug response and resistance mechanisms (Xie et al., 2020; Ding et al., 2021a; Wang et al., 2023; Liu et al., 2018). Accurate circRNA-drug association (CDA) identification is vital for discovering novel therapeutic targets, uncovering drug response mechanisms, and supporting personalized therapy. However, experimental CDA identification is often time-consuming, costly, and inefficient. Thus, developing efficient computational methods is essential to expedite circRNA therapeutic target and drug discovery.

Current deep learning methods have shown remarkable performance in target identification and drug discovery, further advancing CDA prediction. For instance, Deng et al. introduced GATECDA, a graph attention auto-encoder framework for predicting circRNA-drug sensitivity associations (Deng et al., 2022). Yang et al. developed MNGACDA, a graph auto-encoder network that integrates multiview techniques and node-level attention (Yang and Chen, 2023). This model combines multiple information sources from circRNAs and drugs to form a multimodal network, uses multiview techniques to learn low-dimensional embedded representations of circRNAs and drugs, and predicts their association scores through an inner product decoder. Luo et al. presented DPMGCDA, which combines a dual perspective learning mechanism with a pathway masking graph autoencoder to enhance the modeling of circRNA-drug sensitivity relationships (Luo and Deng, 2024). Additionally, Huang et al. proposed DeepHeteroCDA, a computational framework based on a multi-scale heterogeneous network structure and graph attention mechanism, designed to more comprehensively capture the complex sensitivity relationships between circRNAs and drugs (Huang et al., 2025).

In recent years, various innovative drug discovery models have emerged, offering new methodological support and references for circRNA therapeutic target discovery and associated drug development. For example, Zhou et al. proposed the JDASA-MRD model, which combines deep autoencoders and subgraph augmentation to infer microbial responses to drugs (Zhou et al., 2024a). This model captures higher-order neighbor relationships, constrains local message propagation, and enhances drug and microbial representations with remarkable performance. Additionally, Zhou et al. integrated self-supervised strategies and masking mechanisms to explore miRNA responses to small molecule drugs (Zhou et al., 2024b). They also noted that existing drug discovery models often overlook indirectly connected drug-target pairs and proposed a novel model from a global-local perspective (Zhou et al., 2024c). Furthermore, Wei et al. employed multisource prompting with large model technology for efficient drug repurposing (Wei et al., 2024a). Notably, Wei et al. used integrated deep learning to accurately identify unknown drug-target interactions and validated their findings through experiments (Wei et al., 2024b). Although these models do not directly address circRNA therapeutic targets, they provide valuable guidance and references.

Deep learning models like Graph Neural Networks (GNNs) have shown strong performance in CDA prediction tasks, but they also present certain limitations. First, conventional GNNs often rely on shallow feature extraction and struggle to capture higher-order neighbor information, as deeper message propagation may lead to the well-known “oversmoothing” problem. However, higher-order relationships in the circRNA-drug interaction map frequently encode crucial regulatory mechanisms. Second, many existing models depend on complex, task-specific feature engineering pipelines, which can hinder their scalability and generalizability. Moreover, these models tend to overlook the structural semantics of the circRNA-drug graph—namely, the local geometric and topological relationships among CDAs—thereby limiting their ability to model intricate interaction patterns. Recent advances in geometric deep learning have demonstrated that integrating geometric and topological priors, such as curvature, torsion, and simplicial structures, into GNN architectures can enhance representation power in various scientific domains, including protein structure modeling, drug discovery, and biological network analysis. In particular, torsion-based geometric features, which reflect how elements twist or bend in a local topological space, have been shown to encode important structural signals in molecular graphs and manifolds. Despite this progress, their application in the context of circRNA-drug association modeling remains largely unexplored. To address the aforementioned issues, this study proposes a novel graph representation learning framework that explicitly incorporates geometric information to identify potential CDAs. By embedding an analytic torsion technique into the message propagation process over the circRNA-drug graph, our method introduces a new form of local geometric encoding. For each CDA, the framework constructs a local simplicial complex, computes its torsion value, and uses it as an adaptive weight to modulate the message-passing process. This mechanism enhances the model’s capacity to perceive and utilize higher-order geometric and topological cues within the interaction graph, thereby improving its ability to accurately capture potential sensitivity associations between circRNAs and drugs. As a result, our approach contributes to more reliable circRNA therapeutic target discovery and accelerates drug development. Our contributions are summarized as follows:

1) We propose G2CDA, a novel geometric graph representation learning framework for circRNA–drug association (CDA) prediction. Extensive benchmarking demonstrates its strong ability to identify novel CDAs, offering a promising tool to accelerate circRNA-based therapeutic target discovery and drug development.
2) A key innovation of G2CDA is the integration of analytic torsion into the prediction pipeline. By constructing local simplicial complexes and computing torsion values, we transform high-order geometric structures into adaptive, learnable weights—enabling the model to better capture complex circRNA–drug interaction patterns.
3) The computed torsion values are further leveraged as core weights in the message propagation mechanism of the graph neural network. This torsion-guided propagation enhances the model’s focus on geometrically significant substructures, improving representation learning for both circRNAs and drugs.
4) We validate the effectiveness of G2CDA through comprehensive experiments on multiple public datasets, where it consistently outperforms state-of-the-art methods. Case studies demonstrate its potential for translational applications, including the identification of candidate drugs targeting key biomarkers such as ALDH3A2 and ANXA2.

## Materials and methods

This study focuses on identifying potential circRNA–drug associations (CDAs) to facilitate circRNA therapeutic target discovery and drug development. To this end, we propose G2CDA, a novel graph neural network framework that integrates geometric information to capture higher-order interactions between circRNAs and drugs for more accurate CDA prediction. A key innovation of G2CDA is the introduction of the geometric quantity “torsion,” which intuitively reflects the local bending or twisting within the graph structure. By calculating torsion through local simplicial complexes, the model effectively quantifies subtle structural perturbations in the interaction network, analogous to how regulatory influences propagate and modulate biological networks. This approach converts local geometric information into learnable weights, enhancing the model’s ability to capture complex and nuanced relationships between circRNAs and drugs. Furthermore, during message propagation, the torsion information guides both the direction and magnitude of message flow independently of node features, enabling the model to focus on critical structural regions that are most relevant for accurate CDA prediction. Together, these innovations contribute to improved predictive performance. The following sections provide detailed descriptions of the datasets, methodologies, and theoretical principles underpinning this approach.

### Data preparation

This study evaluated the proposed model and comparative models using a publicly available dataset from previous work (Huang et al., 2025). The data, sourced from the circRic database (Vromman et al., 2021), identifies and annotates circRNA molecules across approximately 1,000 human cancer cell lines. The dataset includes experimental data on circRNA responses to drugs, aiming to provide a high-confidence circRNA resource for cancer research and to explore their expression patterns across different cancer types. After screening and removing low-confidence CDAs, 4314 CDAs remained, involving 271 circRNAs and 218 drugs. A circRNA-drug graph was constructed for message propagation. Additionally, 4314 unknown circRNA-drug pairs were randomly selected as negative samples. This balanced sampling approach ensures training process stability.

## Methods

### Model overview

**FIGURE 1:**
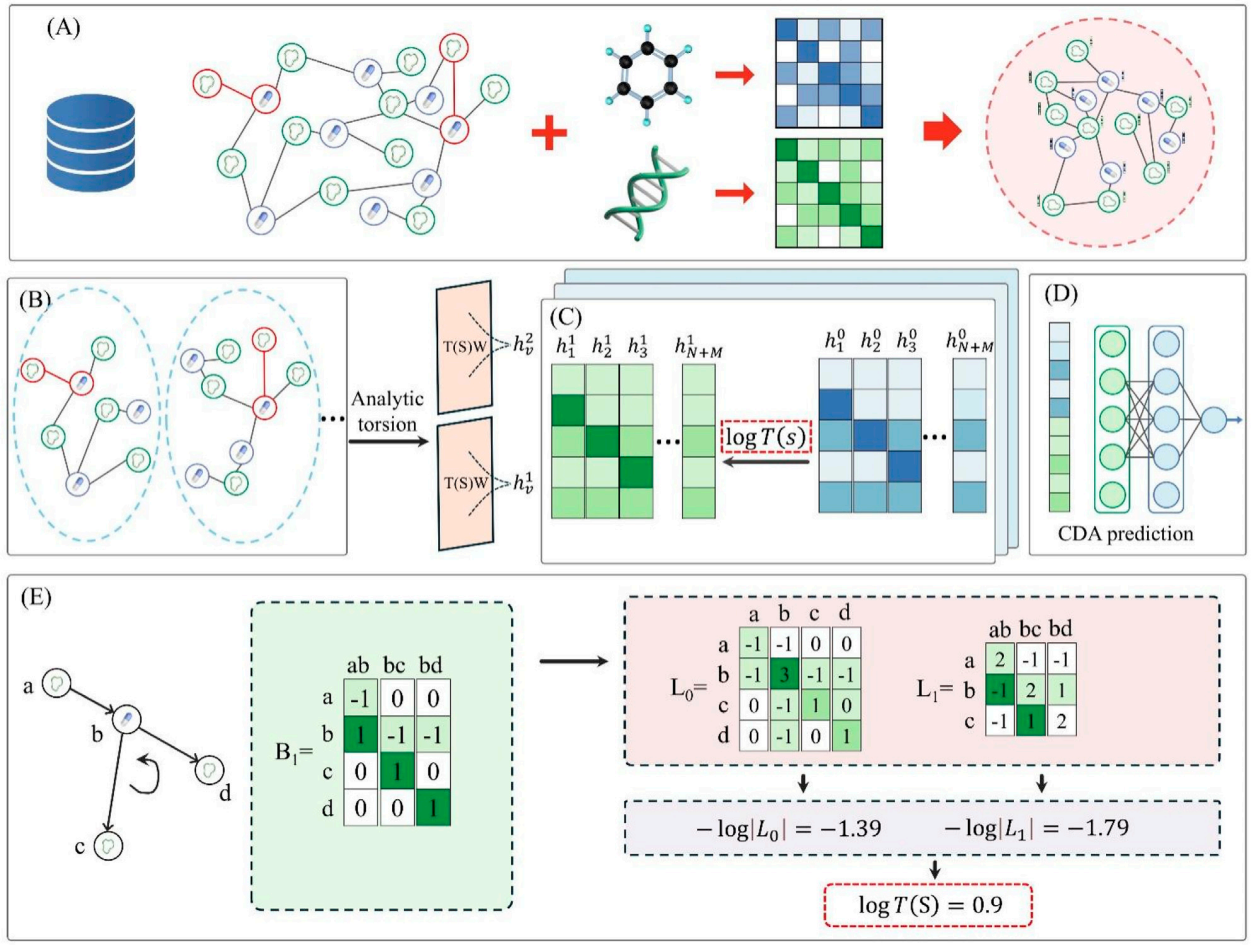
G2CDA’s architecture, comprising: **(A)** construction of the circRNA-drug graph, **(B)** extracting CDA-centered subgraphs, **(C)** calculating CDA torsion, **(D)** integrating geometric message propagation, and **(E)** training and inference.

As shown in Figure 1, the G2CDA model workflow comprises five key modules: constructing the circRNA-drug graph, extracting CDA-centered subgraphs, calculating CDA torsion, integrating geometric message propagation, and training and inference. Module (A) constructs the circRNA-drug graph using known CDAs from the dataset and initial drug/circRNA representations. Module (B) extracts all CDA-centered subgraphs. Module (C) calculates CDA torsion via **1**-simplicial complex. Module (D) fuses these representations to predict drug-circRNA pair scores through an MLP. Module (E) converts CDA torsion into weights for message propagation to refine drug and circRNA representations.

### Problem description

This study first constructs a circRNA-drug graph using known CDAs. Specifically, the graph ***G*** is defined as $G=\langle V_{C}{,V}_{D},E\rangle$ , where $V_{C}{,V}_{D}$ denote the set of circRNAs and drugs nodes, respectively, and E represents the set of observed CDAs. $A\in R^{N\times M}$ is the adjacency matrix of graph ***G***, where ***N*** and ***M*** indicate the number of circRNAs and drugs, respectively. $A_{uv}$ represents the relationship between circRNA ***u*** and drug ***v***, where $A_{uv}=1$ indicates an association and $A_{uv}=0$ indicates no association. The initial representation of a circRNA is defined as $H^{0}=h_{1}^{0},h_{2}^{0},h_{3}^{0},\cdots h_{N}^{0}$ , and that of a drug as $H^{0}=h_{N+1}^{0},h_{N+2}^{0},h_{N+3}^{0},\cdots h_{N+M}^{0}$ . These initial representations can be derived from various sources, such as k-mer frequency characteristics of circRNA sequences and SMILES features of drugs.

Secondly, the study introduces the “analytic torsion” theory, focusing on the local geometric information of CDAs and transforming it into learnable weights to guide message propagation and enhance key region recognition. Specifically, S represents the simplicial complex, with $S_{uv}$ denoting the simplicial complex of edge $A_{uv}$ (the edge between circRNA ***u*** and drug ***v***). Each $A_{uv}=1$ has a corresponding $S_{uv}$ , and $T\left(S_{uv}\right)$ calculates the resolved torque for $S_{uv}$ . Additionally, $Z_{g}$ is the ***g*** *-*th Hodge Laplacian, with $\left|Z_{g}\right|$ representing its determinant. If $Z_{g}$ has zero eigenvalues, the determinant is the product of all non-zero eigenvalues. $B_{g}$ is the boundary matrix corresponding to $Z_{g}$ , and $\gamma_{g}\left(p\right)$ is the regularization function for $Z_{g}$ eigenvalues.

The study aims to enhance message propagation by leveraging the local geometric higher-order information of each CDA based on known CDAs using the “analytic torsion” theory, enabling accurate identification of potential CDAs from unknown circRNA-drug pairs.

### Graph neural network (GNN)

GNN effectively resolves structural information and has been widely applied in bioinformatics, including molecular interaction networks and molecular structure analysis (Cai et al., 2024; Ma et al., 2024; Wang et al., 2024a; Wang et al., 2024b; Wang et al., 2024c; Zhang et al., 2024). This research employs the GNN model to model known CDAs, perform message propagation in the circRNA-drug graph, and output circRNA and drug representations. Specifically, the GNN model’s message propagation involves aggregating and updating information from circRNA and drug nodes. Let $V=V_{C}\cup V_{D}$ , $v_{a}\in V$ , the aggregation process for circRNAs (or drugs) is defined as Equation 1:

$$h_{a}^{i}=f\left(h_{a}^{i-1},\sum_{b\in N\left(a\right)}h_{b}^{i-1},A,\alpha \right)$$ (1)

where $h_{a}^{i}$ represents the representation of node ***a*** at layer ***i***, and f represents the aggregation function. $N\left(a\right)$ is the set of neighbors of node *a*, and α regulates the weights of the neighbors relative to their own information. The GNN model then updates the circRNA (or drug) representation using an activation function and iterates for a set number of times. This study tested the GCN (Kipf and Welling, 2016a), GAT (Veličković et al., 2017), and GIN (Xu et al., 2018) models, incorporating “analytic torque” into CDA prediction tasks.

### Analytic torsion

A simplicial complex S is defined as a collection of multiple simplices. From the total set of circRNAs and drugs, ***g+1*** nodes are selected to form a ***g*** -simplex $\pi^{g}$ , denoted as $v_{0},v_{1},\cdots {,v}_{g}$ . For any $\pi^{g}$ , its non-empty subset is defined as a face, which is the boundary of $\pi^{g}$ when the dimension is ***g*-1**. Geometrically, a *g*-simplex represents the convex hull formed by ***g*+1** nodes. According to this theory, 0-, 1-, 2-, and 3-simplices correspond to vertices, edges, triangles, and tetrahedrons, respectively.

Given an oriented ***S*** with nodes in a predetermined order $v_{0},v_{1},\cdots {,v}_{g}$ , a ***g***-chain is defined as the sum of ***g***-simplices in ***S***, denoted as $d=\sum_{c}\pi_{c}^{g}$ . The set of all ***g***-chains in ***S*** forms the ***g***-chain group $D_{g}$ . A chain complex is formed by integrating a series of chain groups and their corresponding boundary operations: $0\overset{\rho_{0}}{\leftarrow }D_{0}\overset{\rho_{1}}{\leftarrow }D_{1}\overset{\rho_{2}}{\leftarrow }D_{2}\cdots \overset{\rho_{g}}{\leftarrow }D_{g}\cdots$ , where $\rho_{g}:D_{g}\to D_{g-1}$ . The boundary matrix $B_{g}$ is derived from $\rho_{g}$ . The ***g***-th step is defined as $\rho_{g}\pi^{g}=\sum_{j=0}^{g}{\left(-1\right)}^{j}\left[v_{0},v_{1},\cdots ,\hat{v_{j}},\cdots {,v}_{g}\right]$ , where $\hat{v_{j}}$ indicates the removal of $v_{j}$ , and $\rho_{g-1}\rho_{g}=0$ . Let the adjoint operator of $\rho_{g}$ be $\delta^{g}$ . The ***g***-th Hodge Laplacian component of ***S*** is calculated as ${∆}_{g}=\delta^{g}\rho_{g}+\delta^{g+1}\rho_{g+1}$ . Therefore, the ***g***-th Hodge Laplacian matrix of ***S*** is computed as $Z_{g}=B_{g}^{T}B_{g}+B_{g+1}B_{g+1}^{T}$ .

Therefore, in ***S***, the Hodge Laplacian corresponding to the 1-simplex is $Z_{0}=B_{1}B_{1}^{T}$ . Then, the highest-order Hodge Laplacian corresponding to the *m*-simplex is calculated as: $Z_{m}=B_{m}^{T}B_{m}$ . The computation of $Z_{g}$ depends on the simplex relations. For example, when ***g = 0***, $Z_{0}$ can be calculated as Equation 2:

$$Z_{0}\left(u,v\right)=\left\{\begin{array}{l} d\left(\pi_{u}^{0}\right),if\;u=v;\\ -1,if\;u\neq v\;and\;\pi_{u}^{0}⋂\pi_{v}^{0};\\ 0,if\;u\neq v\;and\;\pi_{u}^{0}⋒\pi_{v}^{0}; \end{array}\right.$$ (2)

where $degree\left(\pi_{u}^{0}\right)$ denotes the degree of $\pi_{u}^{0}$ , $\pi_{u}^{0}⋂\pi_{v}^{0}$ indicates that simplices $\pi_{u}^{0}$ and $\pi_{v}^{0}$ are upper adjacent, and $\pi_{u}^{0}⋒\pi_{v}^{0}$ denotes non-upper adjacency. Furthermore, the value of $Z_{g}\left(u,v\right)$ can be inferred based on geometric information such as upper adjacency, lower adjacency, and directional consistency between two ***g***-simplices (Shen et al., 2025). Specifically, on the circRNA-drug graph, this can be treated as known geometric information corresponding to CDAs.

### Analytical torsion calculation

$Z_{g}$ is a positive semi-definite matrix with eigenvalues $\left\{\theta_{j}\right\}$ . Its regularization function $\gamma_{g}\left(p\right)$ is computed as $\gamma_{g}\left(p\right)=\sum_{\theta_{j}>0}\;\frac{1}{\theta_{j}^{p}}$ , where p is the regularization factor. Then, the analytical torsion for ***S*** can be calculated in logarithmic form as follows:

$$\log\;T\left(S\right)=\frac{1}{2}\sum_{g=0}^{N+M}{\left(-1\right)}^{g}g\gamma_{g}^{\left(1\right)}\left(0\right)$$ (3)

where $\gamma_{g}^{\left(1\right)}\left(0\right)$ represents the first derivative of $\gamma_{g}\left(p\right)$ evaluated at p=0 . Assuming the determinant of $Z_{g}$ is the product of its non-zero eigenvalues $\left|Z_{g}\right|=\prod_{\theta_{j}>0}\theta_{j}$ , then $\gamma_{g}^{\left(1\right)}\left(0\right)=-\log\;\left|Z_{g}\right|$ . Equation 3 can thus be further transformed as follows:

$$\log\;T\left(S\right)=\frac{1}{2}\sum_{g=0}^{N+M}{\left(-1\right)}^{g+2}glog\;\left|Z_{g}\right|$$ (4)

For the **1**-dimensional simplicial complex **S** of the circRNA-drug graph, its analytic torsion $T\left(S\right)$ is calculated as $T\left(S\right)={\left|Z_{1}\right|}^{\frac{1}{2}}$ , where $Z_{1}=B_{1}^{T}B_{1}$ . For the **2**-dimensional simplicial complex, the analytic torsion is computed as $T\left(S\right)=\frac{{\left|Z_{1}\right|}^{\frac{1}{2}}}{\left|Z_{2}\right|}$ . The calculation of analytic torsion depends on the simplicial complex, and the specific process is illustrated in Figure 1E.

### Analytic torsion on CircRNA-Drug network

This study incorporates analytic torsion calculations into message propagation on the circRNA-drug graph, based on previous work (Reidemeister, 1935). Within the GNN model, analytic torsion is transformed into CDA weights for message aggregation. Equation 4 is formalized as:

$$h_{a}^{i}=sigmod\left(\sum_{b\in N\left(a\right)⋃a}\frac{1}{\sqrt{d\left(a\right)}\sqrt{d\left(b\right)}}\left|\log\;T\left(S_{a,b}\right)\right|Wh_{b}^{i-1}\right)$$ (5)

where $sigmod\left(\;\right)$ is the activation function, $N\left(a\right)$ represents the neighbors of node ***a***, and $d\left(\;\right)$ indicates node degree. $h_{b}^{0}$ corresponds to the initial representation of the ***b***-th node on the circRNA-drug graph, defined as the ***b***-th row of matrix **A**.

In Equation 5, $S_{a,b}$ refers to the local simplicial complex constructed from CDA ***<a, b>***. To determine the local simplicial complex, one common approach is to extract a subgraph from direct neighbors. This subgraph includes nodes **a, b**, all their neighbors, and all edges (CDAs) between circRNAs and drugs within this group. A second-order subgraph can be derived from the second-order neighbors of circRNAs (or drugs). For a **2**-dimensional 2-simplex complex $S_{a,b}$ , a triangle should be constructed from three nodes. The model can incorporate higher-order neighbors to enhance local geometric information. If any circRNA and drug within the subgraph are associated, ***g+1*** entities can form a ***g***-simplex. The computation of $S_{a,b}$ involves the dimension ***Q*** of the simplicial complex and the subgraph order ***P***. When ***Q*** and ***P*** are both 1, the message propagation process on the circRNA-drug graph is illustrated in Figure 1E.

### CDA prediction

This study integrates GNN models (e.g., GCN, GAT, GIN) for torque analysis on the circRNA-drug graph. Once the models complete the preset number of message propagation processes, the final representations of circRNAs and drugs are extracted. The study aims to calculate the probability of edges (associations) between circRNAs and drugs based on these representations. Let the final representations of circRNA ***u*** and drug ***v*** be $h_{u}$ and $h_{v}$ , respectively. The edge probability for the $\langle u,v\rangle$ pair is predicted as Equation 6:

$${\hat{q}}_{xy}=MLP\left(∥\left(h_{u}+h_{v},h_{u}⊙h_{v},h_{u}∥h_{v}\right)\right)$$ (6)

where MLP denotes a multilayer perceptron, ∥ represents concatenation, and ⊙ denotes the Hadamard product. The study employs the binary cross-entropy (BCE) function to compute the final loss.

## Results

### Performance comparison

This study systematically evaluates the G2CDA model’s performance in CDA prediction through comprehensive comparison experiments. The experimental design includes multiple benchmark model comparisons. For CDA prediction, the study selects several state-of-the-art models: GATECDA (Deng et al., 2022), MNGACDA (Yang and Chen, 2023), DPMGCDA (Luo and Deng, 2024), and DeepHeteroCDA (Huang et al., 2025). These models represent the current best practices in CDA prediction. Additionally, other GNN-based interaction prediction models are included, such as variational graph auto-encoders (VGAE) (Kipf and Welling, 2016b), Multi-view variational graph auto-encoder with matrix factorization (VGAMF) (Ding et al., 2021b), graph convolutional network based GCNMDA (Long et al., 2020), and local attention graph convolutional network (LAGCN) (Yu et al., 2021). Although these models have not been previously applied to CDA prediction, they have demonstrated success in other association prediction tasks. Furthermore, five classical machine learning algorithms are selected as benchmark references: Support Vector Machines (SVM), Random Forest (RF), K Nearest Neighbors (KNN), Extreme Gradient Boosting (XGBoost), Adaptive Boosting (AdaBoost) and Multilayer Perceptron (MLP). These algorithms, widely used in supervised learning, provide a performance comparison at the traditional method level. The multi-dimensional comparison system ensures the evaluation results are both comprehensive and reliable by covering both domain-specific and generalized models. Following established methodologies (Wang et al., 2024d; Wei et al., 2024c; Feng et al., 2025; Fu et al., 2025; Chen et al., 2024), we adopted five key metrics: AUC (Area Under the Curve), AUPR (Area Under the Precision-Recall Curve), Accuracy, Recall, and F1-score.

According to Table 1, the G2CDA model outperforms all comparative methods across all evaluation metrics, achieving an AUC of 94.91%, AUPR of 94.48%, F1-score of 87.06%, accuracy of 87.70%, and recall of 83.03%. This demonstrates its superior prediction ability and robustness. Traditional machine learning methods like SVM, RF, KNN, XGBoost, and AdaBoost struggle to effectively model graph structures and complex nonlinear relationships, resulting in inferior overall performance. While generic GNN models such as VGAE, VGAMF, GCNMDA, and LAGCN can capture some structural information, they lack specific optimization for CDA prediction, leading to a significant performance gap. Among task-specific models, DeepHeteroCDA, MNGACDA, and DPMGCDA show relatively strong performance, particularly DeepHeteroCDA with an AUC of 92.33%, but they still underperform the G2CDA model across key metrics. Overall, the G2CDA model excels not only in prediction accuracy but also in handling sample imbalance, structural modeling, and multimodal information fusion, confirming its effectiveness for CDA prediction tasks.

**TABLE 1:** Comparison results between G2CDA and other advanced models (%).

Methods	AUC	AUPR	F1-score	Accuracy	Recall
SVM	86.48	85.47	80.49	79.28	85.50
RF	88.81	88.85	82.04	81.65	83.83
KNN	86.42	87.60	79.26	79.01	80.20
XGBoost	89.97	89.96	82.94	82.52	84.94
AdaBoost	88.52	88.88	82.07	81.55	84.43
VGAE	86.28	87.30	79.88	78.92	82.27
VGAMF	87.40	86.62	81.76	81.04	84.37
GCNMDA	87.78	87.62	81.98	81.19	84.28
GATECDA	88.46	89.29	81.94	81.68	83.16
LAGCN	89.82	90.23	82.85	82.61	84.03
MNGACDA	90.98	91.50	84.13	83.79	85.92
DPMGCDA	90.15	91.73	84.08	84.24	83.25
DeepHeteroCDA	92.33	92.93	85.61	85.20	88.07
MLP	93.53	93.09	86.74	86.59	86.63
G2CDA	94.91	94.48	87.06	87.70	83.03

### Performance evaluation

In Table 2, the five - fold cross - validation results show that the G2CDA model has excellent stability and generalization in CDA prediction. The model’s average AUC is 94.58% (±0.53%), and average AUPR is 94.02% (±0.43%), indicating its strong discriminative ability comparable to top existing models. Notably, the F1 score (85.97% ± 1.26%) and accuracy (86.67% ± 1.15%) show fluctuations of less than 1.5 standard deviations, demonstrating the model’s robustness to data distribution changes. In Fold 1 and Fold 3, the AUC reaches 94.91% and 94.93%, respectively, with the F1 score surpassing 87%. The recall rate, while fluctuating between 79.69% and 83.03%, remains consistently above 80%, highlighting the model’s effectiveness in retaining positive sample features via its torsion - weight - guided information dissemination mechanism. These findings confirm that the local - simple - complex - shape - based torsion counting strategy enhances the model’s geometric interaction feature capture ability, and the non - original - feature - dependent attention propagation mechanism offers significant advantages for modeling complex biological relationships. This work provides a novel methodological framework for CDA prediction.

**TABLE 2:** Results of 5-fold cross validation of G2CDA model (%).

Folds/metrics	AUC	AUPR	F1-score	Accuracy	Recall
1	94.91	94.48	87.06	87.7	83.03
2	93.74	93.63	84.33	85.09	79.69
3	94.93	94.49	87.11	87.76	82.90
4	93.93	93.55	84.81	85.68	79.95
5	94.38	93.95	86.53	87.11	82.60
Average	94.58 ± 0.53	94.02 ± 0.43	85.97 ± 1.26	86.67 ± 1.15	81.63 ± 1.67

### Parameter experiments

This study examines various factors, including GNN encoder types, GNN layer numbers, hidden layer dimensionalities, output layer dimensionalities and node initial representations, necessitating multiple parameter experiments to verify the G2CDA model’s stability and establish parameter setting guidelines.

Different GNN models were analyzed for their information transfer and feature extraction capabilities. Three representative GNN variants were experimented with to adapt to diverse graph structures and task requirements. GCN, leveraging Laplacian smoothing, suits homogeneous graphs. GIN, with multilayer perceptron-like expressiveness, handles complex heterogeneous graphs. GAT introduces attention mechanisms to enhance sensitivity to key connections. Comparative experiments among these encoders confirmed their performance and adaptability under different graph encoding approaches.

Table 3 evaluates their performance across metrics like AUC, AUPR, F1-score, Accuracy, and Recall. GCN showed the best overall performance with an AUC of 94.91%, AUPR of 94.48%, F1 score of 87.06%, and accuracy of 87.70%. GIN had slightly lower accuracy (86.72%), AUC (94.45%), and AUPR (94.06%), with an F1 score of 85.71% and a significantly lower Recall (79.58%), indicating weaker positive sample recall. GAT achieved the highest Recall (88.05%), suggesting its attention mechanism aids positive sample recognition, but had lower AUC (93.90%) and AUPR (93.41%), showing shortcomings in overall discriminative ability and stability. In summary, GCN balanced all five metrics best, maintaining high accuracy and F1 scores while ensuring good recall and generalization, making it the optimal encoder choice. GAT’s high recall also offers a feasible alternative for applications requiring high positive sample sensitivity.

**TABLE 3:** Results of G2CDA with different GNN encoders (%).

Models/metrics	AUC	AUPR	F1-score	Accuracy	Recall
GCN	94.91	94.48	87.06	87.70	83.03
GIN	94.45	94.06	85.71	86.72	79.58
GAT	93.90	93.41	87.15	86.98	88.05

**FIGURE 2:**
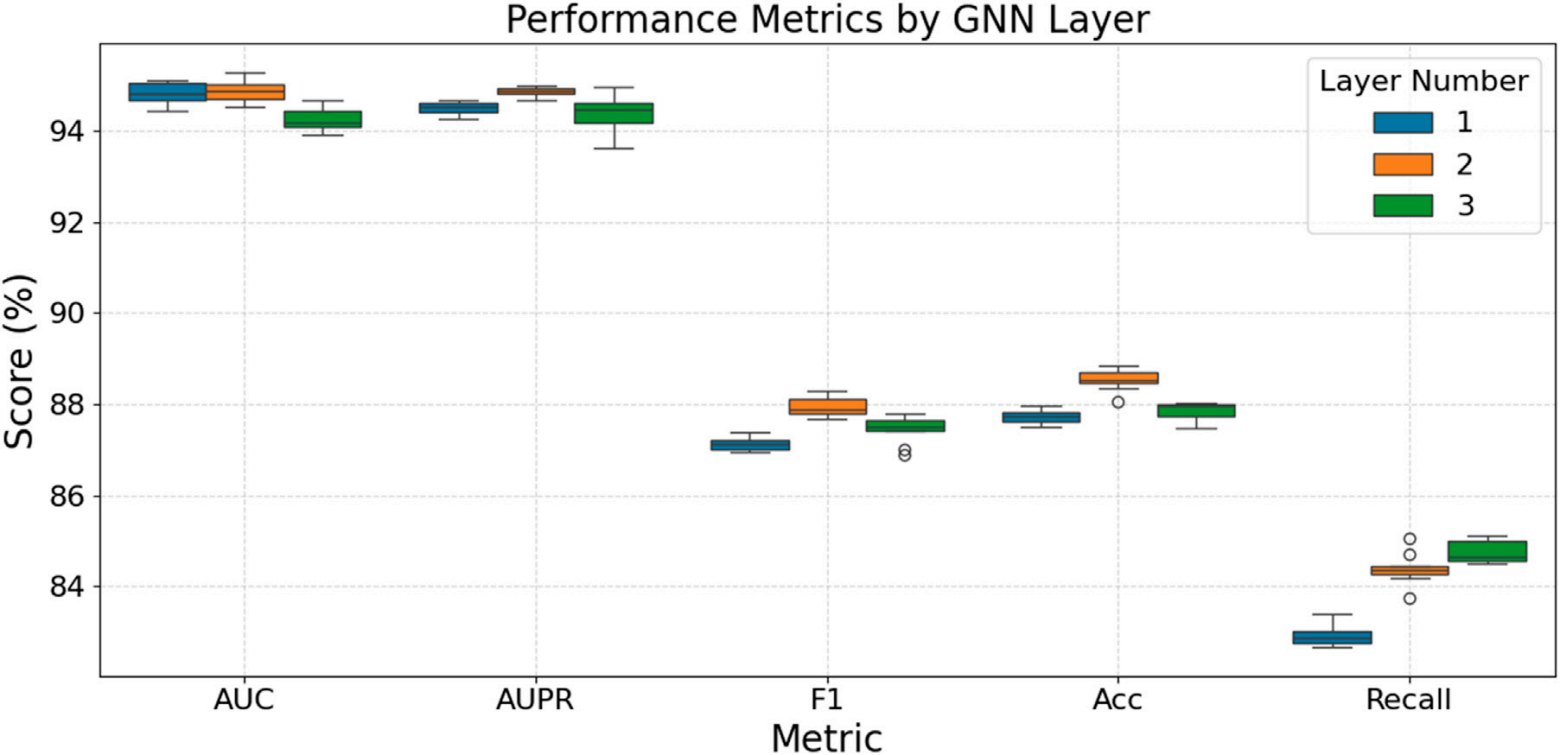
Model performance at different GNN layers.

Figure 2 assesses the impact of different GNN layer depths on model performance. Overall, a 2-layer GNN architecture demonstrates the optimal balance and stability across these metrics, achieving the highest median scores for AUPR, F1-score, and Accuracy, while also exhibiting strong and stable performance in Recall. In contrast, although a 1-layer GNN performs best on the AUC metric, it shows a notable deficiency in Recall and is generally not the optimal choice for F1-score and Accuracy. Increasing the GNN layers to three does not yield significant performance improvements across most metrics; instead, it may lead to a slight decrease in some indicators (such as AUC) or exhibit greater volatility and instability in metrics like F1-score and Recall, even though its median Recall is marginally higher than that of the 2-layer model. Thus, the 2-layer architecture optimally balances performance, supporting the “shallow geometry perception” design concept.

**FIGURE 3:**
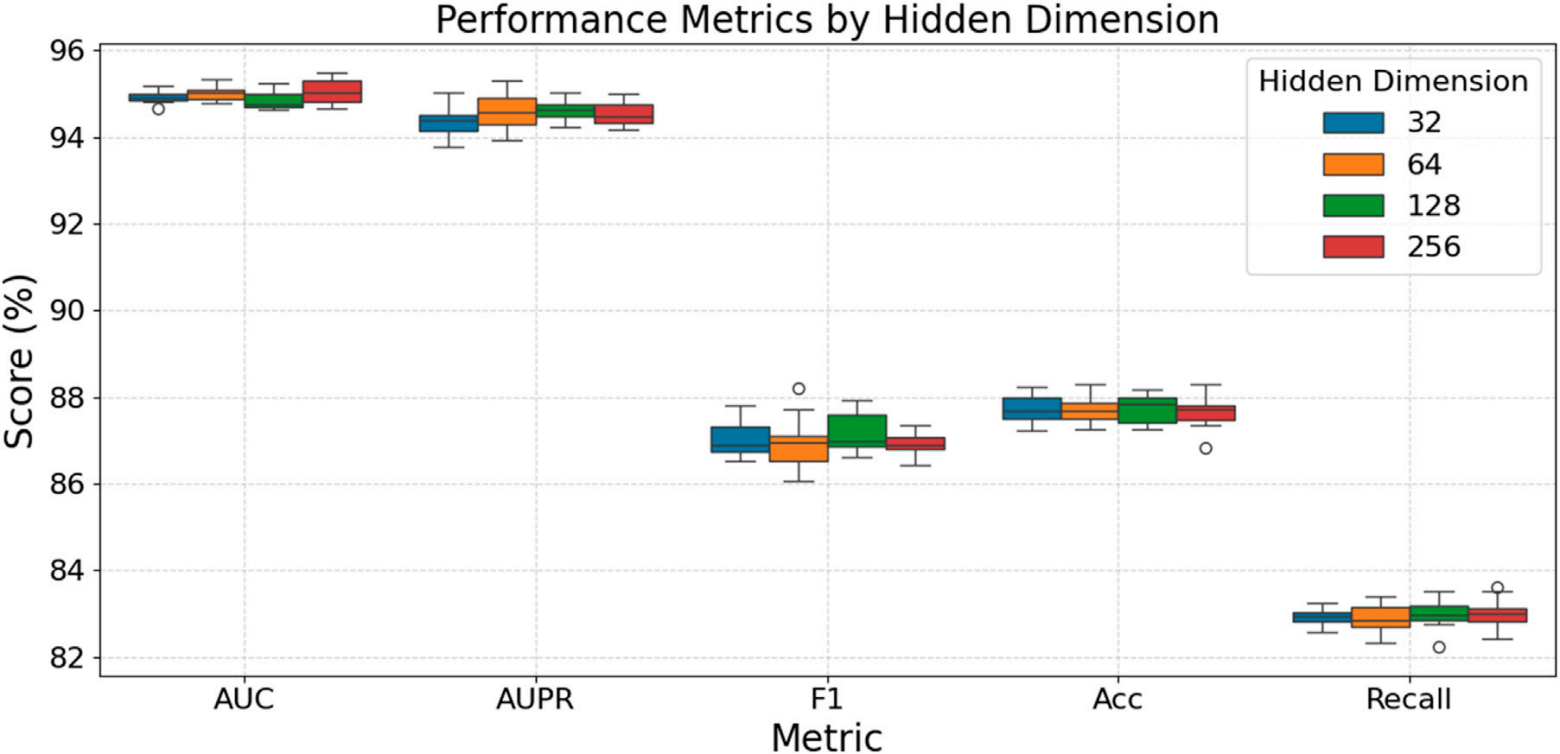
Model performance at different hidden layer dimensions.

Figure 3 examines how different GNN hidden layer dimensionalities affect model performance. As depicted, for the AUC and AUPR metrics, all tested hidden dimensions exhibit very similar high performance, with scores generally concentrated between 94% and 95%, indicating that hidden dimension size has a minimal effect on these two metrics, although dimensions 64, 128, and 256 show slightly better stability for AUPR. However, for F1-score and Accuracy, a hidden dimension of 128 appears to provide the optimal performance, with median F1-scores around 87.5% and median Accuracy scores approaching 87.8%–87.9%. Regarding Recall, while there is a slight upward trend in median scores with increasing dimensionality (around 83.1% for the 256 dimension), this is accompanied by greater volatility and the appearance of some outliers, particularly at the 256 dimension. In summary, while larger hidden dimensions do not significantly alter AUC or AUPR performance, a mid-range dimension like 128 seems to offer the best balance for F1-score and Accuracy, whereas dimensions 64 and 128 also provide good and stable performance for Recall, avoiding the increased variance potentially introduced by the largest dimension.

**FIGURE 4:**
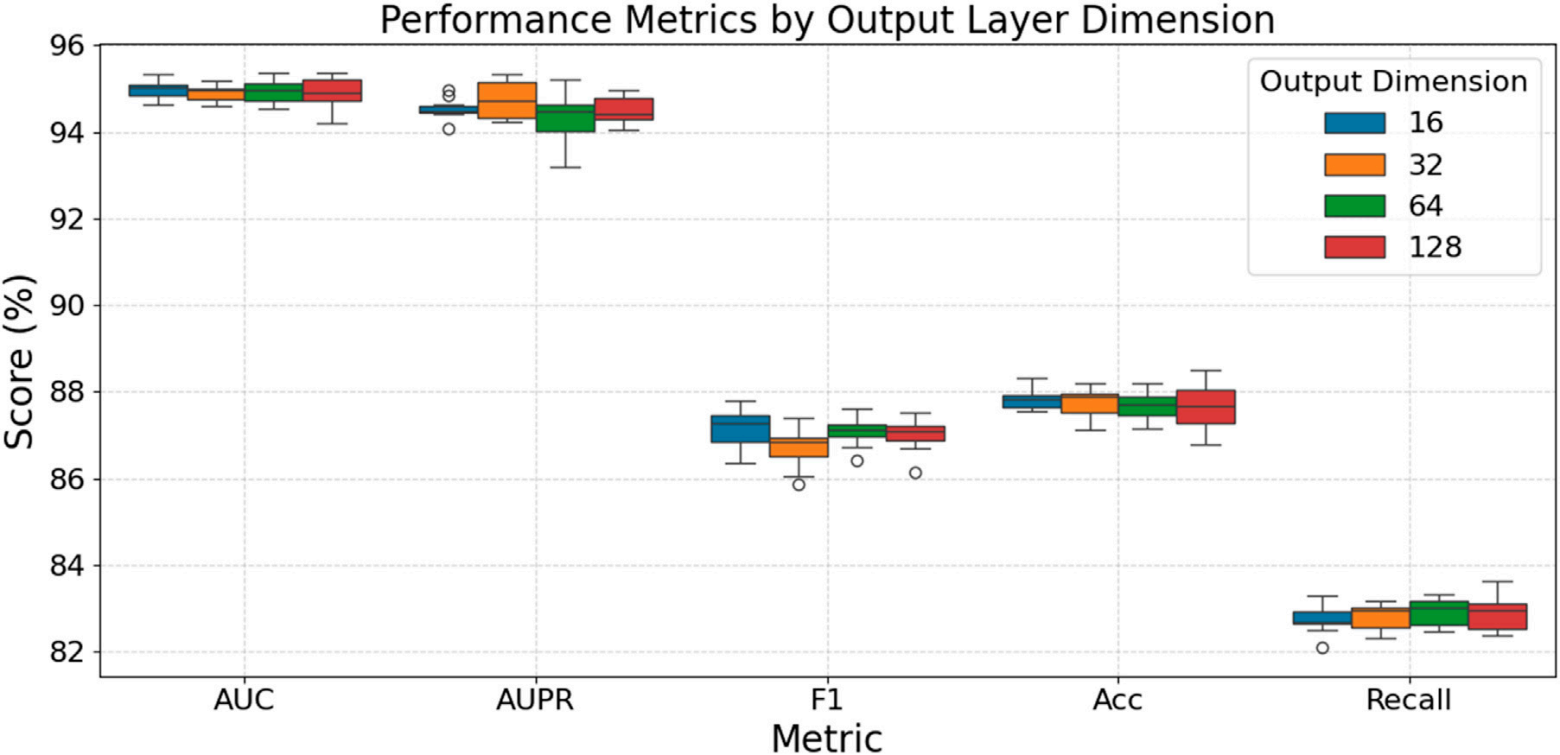
Model performance at different output layer dimensions.

Figure 4 evaluates how varying the output layer dimension impacts model performance. For AUC, Accuracy, and Recall, the choice of output dimension within this range appears to have a minimal impact, with all tested dimensions yielding very similar and stable high scores; for instance, AUC scores consistently hover around 94.8%–95%, and Accuracy scores are tightly grouped around 87.6%–87.8%. In the case of AUPR, dimensions 32 and 128 demonstrate slightly more stable and marginally higher median performance (around 94.5%–94.8%) compared to dimensions 16 and 64. For the F1-score, an output dimension of 32 results in a slightly lower median and increased variability, while dimensions 16, 64, and 128 offer comparable and slightly better median performance (around 87.0%–87.2%). Overall, while most metrics show little sensitivity to the output layer dimension, dimensions such as 32 or 128 might provide minor advantages in AUPR stability, and dimensions other than 32 could be preferable for F1-score, but generally, the model’s performance is robust across these output dimension variations.

In the CDA modeling task, circRNA and drug features play a crucial role in the GNN’s ability to understand semantic and graph structures. To evaluate the impact of different feature representations on model performance, we use various node feature construction methods in our experiments. These include Uniform and Normal distributions for random initialization, which are suitable for scenarios with no prior knowledge and mainly test the model’s structural learning ability. One-hot encoding assigns unique identities to nodes, distinguishing them without providing semantic information. Sim features, based on the circRNA-drug similarity matrix, leverage domain-specific prior knowledge to enhance the model’s perception of actual associations. CircRNA similarity is quantified using two metrics: sequence-based similarity and GIP (Gaussian interaction profile) kernel similarity (Kipf and Welling, 2016a; Veličković et al., 2017). Similarly, drug similarity is evaluated from two perspectives:structural similarity and GIP kernel similarity. The comprehensive similarity matrices for circRNAs and drugs are obtained by merging the respective similarity matrices. Position features use the adjacency matrix to represent nodes’ positions in the graph structure, emphasizing the role of structural connectivity relationships.

**FIGURE 5:**
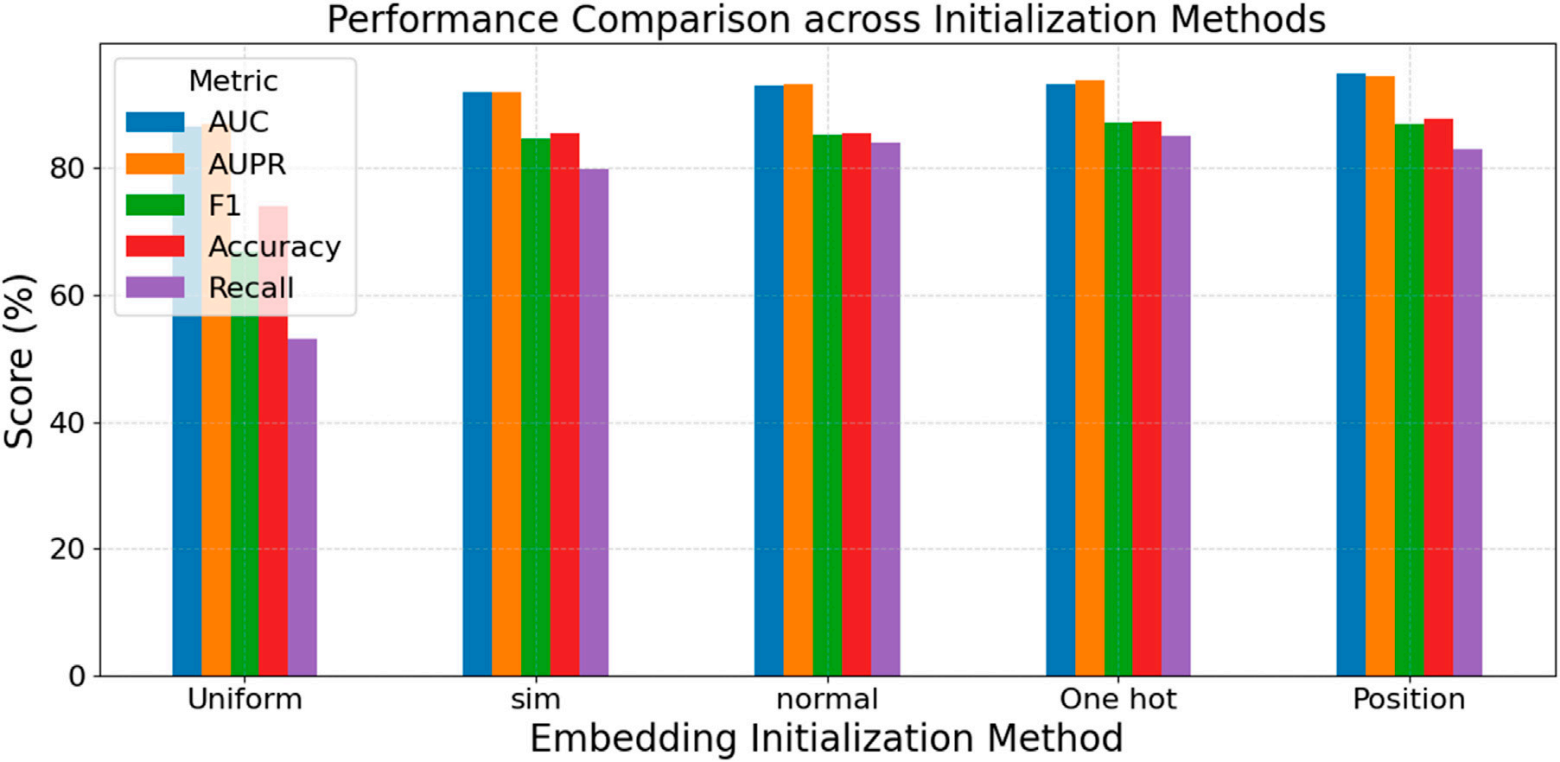
Performance comparison across initialization methods.

The experimental results in Figure 5 demonstrate the crucial role of node features in GNN models. Position features (using the adjacency matrix) achieve the best overall performance, with the highest AUC (94.91%), AUPR (94.48%), and Accuracy (87.70%), indicating that structural information significantly impacts model accuracy and stability. One-hot encoding and Normal initialization also show strong performance, particularly in F1 and Recall. The F1 score of One-hot reaches 87.17%, suggesting that clearly distinguishing node identities aids the model’s discriminative ability. In contrast, Uniform initialization performs the weakest across all metrics, especially with a Recall of only 53.09%, indicating a lack of effective structural or semantic information that limits the model’s learning ability. Sim features, based on biomolecular similarity, perform better than random initialization but not as well as Position features, indicating that domain knowledge can enhance the model’s representation and generalization capabilities.

In summary, incorporating structural information (e.g., adjacency matrix) and semantic similarity (e.g., Sim features) is vital for improving graph neural network performance. Simple random initialization struggles to achieve excellent performance without prior knowledge. These findings provide important guidance for future model design and feature construction.

### Ablation experiments

The resolving torsion module, a core innovative component of our model, significantly enhances the representation of graph structures. It captures higher-order node relationships by modeling local graph regions with simplicial complexes. We further integrate topological torsion values into edge weights, strengthening the model’s sensitivity to local geometric and topological features. This method boosts the graph neural network’s modeling dimensions and improves its expressiveness and discriminative power in complex graph data.

**FIGURE 6:**
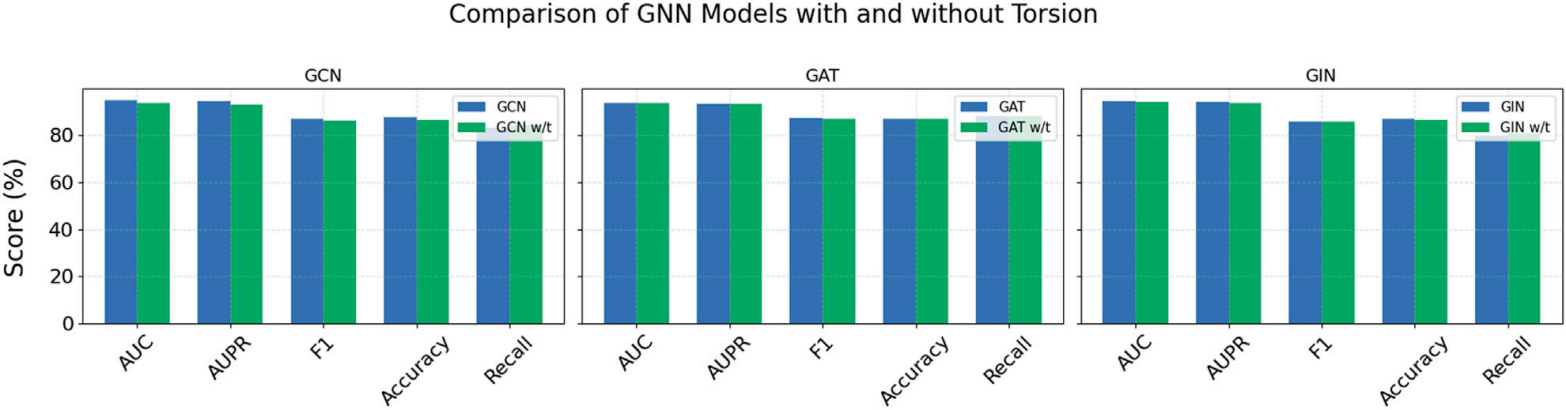
Comparison of GNN models with and without Torsion (“w/t” refers to the model without the “Torsion” component).

To validate this model’s contribution, we designed an ablation study. By removing torsion weights or replacing them with simple edge similarity metrics, we observed performance changes across multiple evaluation metrics. Results in Figure 6 show that the torsion module substantially improves overall model performance. For example, in GCN models, AUC increases from 93.64% to 94.91%, AUPR from 93.11% to 94.48%, and F1 from 86.29% to 87.06%. These improvements highlight how torsion-derived topological information enhances the model’s discriminative and generalization abilities. Although GAT and GIN models show minor changes in Recall after incorporating torsion, their F1 scores and accuracy improve, indicating torsion weights play a key role in capturing local structural differences and enhancing feature expression.

In summary, the torsion module is crucial for improving the model’s ability to understand and utilize higher-order graph relationships, making it a key factor in enhancing model performance.

### Case analysis

To validate the constructed model’s real - world biomedical applicability, a case study was conducted. circRNAs were selected from databases, and their potential drug interactions were predicted using the trained model. This assessed the model’s accuracy in identifying circRNA - drug associations and its utility in studying disease mechanisms and screening new drugs.

ALDH3A2 (aldehyde dehydrogenase 3 family member A2), a key human enzyme encoding fatty aldehyde dehydrogenase, is mainly found in peroxisomes and the endoplasmic reticulum. It oxidizes medium - and long - chain aliphatic aldehydes to fatty acids, playing a crucial role in lipid metabolism and cellular antioxidant responses (Rizzo and Carney, 2005). Highly expressed in liver, skin, and brain tissues, it is vital for skin barrier function and nervous system performance. ALDH3A2 loss - of - function or mutation can cause Sjögren - Larsson syndrome, an autosomal recessive disorder with symptoms like ichthyosis, mental retardation, and spastic paralysis. Using the trained model, drugs related to ALDH3A2 were predicted, and the top 20 candidates with the highest model evaluation scores are listed in Table 4.

**TABLE 4:** Top 20 predicted drugs with potential associations with ALDH3A2.

Drugs	Evidence	Drugs	Evidence
SNX-2112	Confirmed	YK 4–279	Confirmed
MP470	Confirmed	BX-795	Confirmed
CP724714	Confirmed	GSK690693	Confirmed
Axitinib	Confirmed	CH5424802	Confirmed
CCT018159	Confirmed	Navitoclax	Confirmed
AMG-706	Confirmed	TPCA-1	Confirmed
KIN001-055	Confirmed	BHG712	Confirmed
Cetuximab	Confirmed	XMD8-92	Confirmed
UNC0638	Confirmed	Temozolomide	Unconfirmed
Olaparib	Confirmed	Embelin	Unconfirmed

ANXA2 (Annexin A2), a calcium-dependent phospholipid-binding protein, is widely expressed in various human tissues, particularly in epithelial and vascular endothelial cells (Wang et al., 2019). Located in the cytoplasm, inner cell membrane, and extracellular environment, it participates in processes like membrane transport, cytoskeletal remodeling, and fibrinolytic system regulation. ANXA2 also acts as a co-receptor for tissue fibrinogen activator and fibrinogen, influencing fibrinogenesis and thrombolysis. Its abnormal expression is linked to diseases, especially cancers, where it is associated with tumor invasion, metastasis, and drug resistance, making it a potential tumor marker and therapeutic target. Additionally, ANXA2 plays roles in viral infections, autoimmune diseases, and inflammatory responses, highlighting its broad biomedical and clinical significance. Using the trained model, we predicted drugs related to ANXA2, and the top 20 candidates based on model evaluation scores are listed in Table 5.

**TABLE 5:** Top 20 predicted drugs with potential associations with ANXA2.

Drugs	Evidence	Drugs	Evidence
Camptothecin	Confirmed	Midostaurin	Confirmed
Bortezomib	Confirmed	CP724714	Confirmed
Tipifarnib	Confirmed	CHIR-99021	Confirmed
Salubrinal	Confirmed	YM201636	Confirmed
Tamoxifen	Confirmed	Afatinib	Confirmed
Vinblastine	Confirmed	GSK429286A	Confirmed
KIN001-055	Confirmed	GSK690693	Confirmed
Embelin	Confirmed	Ruxolitinib	Confirmed
Docetaxel	Confirmed	Cetuximab	Confirmed
Nilotinib	Confirmed	Gemcitabine	Unconfirmed

ASPH (Aspartate Beta-Hydroxylase) is a protein encoded by the human ASPH gene and belongs to the 2 - ketoglutarate - dependent dioxygenase family (Hou et al., 2018). It plays a role in various biological processes, particularly in cell migration and cancer. Using the trained model, we predicted drugs related to ASPH. The top 20 candidates with the highest model evaluation scores are listed in Table 6.

**TABLE 6:** Top 20 predicted drugs with potential associations with ASPH.

Drugs	Evidence	Drugs	Evidence
Elesclomol	Confirmed	Zibotentan	Confirmed
Temsirolimus	Confirmed	Gemcitabine	Confirmed
KIN001-055	Confirmed	TAK-715	Confirmed
Parthenolide	Confirmed	Embelin	Confirmed
CCT018159	Confirmed	Vinblastine	Confirmed
Temozolomide	Confirmed	Tipifarnib	Confirmed
Tamoxifen	Confirmed	Dasatinib	Confirmed
Salubrinal	Confirmed	CEP-701	Confirmed
AS605240	Confirmed	Midostaurin	Confirmed
BIRB 0796	Confirmed	Bicalutamide	Unconfirmed

CALD1 (Caldesmon 1), encoded by the CALD1 gene, is a human-encoded regulatory protein related to actin and is expressed in various tissues, particularly functional in smooth muscle and non-myocytes (Cheng et al., 2021). It plays key roles in cytoskeletal remodeling, cell migration, and muscle contraction, making it crucial in multiple biological processes, especially in cancer. We used our trained model to predict drugs related to CALD1, and the top 20 drug candidates based on model evaluation scores are listed in Table 7.

**TABLE 7:** Top 20 predicted drugs with potential associations with CALD1.

Drugs	Evidence	Drugs	Evidence
AS605240	Confirmed	Nilotinib	Confirmed
Camptothecin	Confirmed	KIN001-055	Confirmed
Bortezomib	Confirmed	Docetaxel	Confirmed
CP724714	Confirmed	selumetinib	Confirmed
S-Trityl-L-cysteine	Confirmed	Salubrinal	Confirmed
17-AAG	Confirmed	Epothilone B	Confirmed
Tipifarnib	Confirmed	Axitinib	Confirmed
CI-1040	Confirmed	Temsirolimus	Confirmed
PD-0325901	Confirmed	PD-173074	Unconfirmed
Ruxolitinib	Confirmed	Embelin	Unconfirmed

The experimental results regarding the four specific circRNAs showed that most drug associations predicted by the model were validated in existing databases, confirming the model’s accuracy and reliability in identifying potential circRNA-drug relationships. These findings not only enhance the model’s applicability to real-world data but also validate its potential biomedical applications, particularly in circRNA function research and drug discovery.

## Conclusion

To address the computational demands of the proposed geometric modeling approach, an empirical evaluation was performed to quantify the time necessitated by local structural extraction and the computation of torsion-based geometric features. The results indicate that the initial phase, involving the extraction of 1-hop, 2-hop, and 3-hop subgraph structures for each circRNA-drug pair, is relatively efficient, requiring approximately 0.326 s per pair. However, the subsequent phase of geometric feature computation, which includes the construction of simplicial complexes and the calculation of torsion features, demands a significantly greater processing time, averaging approximately 905 s. This substantial increase in computational duration underscores the considerable overhead associated with the incorporation of higher-order geometric information. While this computational cost is considered acceptable for datasets of moderate size, particularly given the concomitant improvements in predictive performance, it may pose scalability challenges when the model is applied to large-scale or real-time biomedical knowledge graphs. Consequently, future research will focus on exploring more efficient approximation techniques or parallelization strategies to reduce the time required for geometric encoding.

circRNA plays a crucial regulatory role in the cellular microenvironment, with its aberrant expression frequently linked to disease progression, including cancer. Additionally, circRNA is widely involved in regulating cellular drug resistance, making the study of circRNA–drug associations (CDAs) vital for therapeutic target discovery and drug development. While existing graph neural network-based methods have advanced CDA prediction, they often overlook important geometric information inherent in the local structures around known CDAs, which limits their predictive power. In this study, we propose a novel graph representation learning framework that integrates geometric information through a resolved torsion technique. By identifying each CDA’s local simplicial complex and computing its torsion value as an edge weight during message propagation, our model effectively captures higher-order geometric interactions within the circRNA–drug network. This innovation enables enhanced perception of subtle structural perturbations, thereby improving the accuracy of unknown CDA identification beyond existing methods. Despite these advancements, the model has limitations. For example, the current approach may face challenges in scalability when applied to extremely large or heterogeneous datasets, and the reliance on local simplicial complexes assumes sufficient data quality and completeness. Future work will focus on extending the model’s applicability to broader datasets, integrating multi-omics information, and exploring adaptive geometric encoding strategies to further enhance predictive performance. Overall, our findings demonstrate that incorporating torsion-based geometric information is a promising direction for advancing circRNA–drug association prediction, with potential to accelerate therapeutic target discovery and drug development.

## Data Availability

The original contributions presented in the study are included in the article/supplementary material, further inquiries can be directed to the corresponding authors.
